# Editorial Comment: Dorsal oral mucosa graft in combination with ventral penile flap as an alternative to repair obliterative stenosis of the anterior urethra in a single surgical time

**DOI:** 10.1590/S1677-5538.IBJU.2019.0299.1

**Published:** 2020-01-13

**Authors:** Luciano A. Favorito

**Affiliations:** 1 Unidade de Pesquisa Urogenital - Universidade Estadual do Rio de Janeiro - Uerj, Rio de Janeiro, RJ, Brasil;; 2 Hospital Federal da Lagoa, Rio de Janeiro, RJ, Brasil

Recently we comment in International Brazilian Journal of Urology a important paper about the use of penile skin fap in anterior penile urethral stricture (1, 2). In this previous paper Dr. Hmida shows that penile skin ap is easy to perform, do not need urethral mobilization and had a success rate of 88%. In the present paper, Dr. Giudice in a interesting prospective study with the use in a single procedure the combination of dorsal onlay oral mucosa graft and ventral fasciocutaneous penile skin flap in in 21 patients with long segment urethral obliteration and shows that in only 14% of the patients the procedure failed. There are several options for the treatment of anterior urethral stricture (2–4). For long anterior strictures the use of grafts is very usual (4–6). The buccal mucosa grafts and fascio-cutaneous flaps are frequently used in long anterior urethral strictures (2, 7). In this paper the authors shows that the combination of the two methods (dorsal onlay oral mucosa graft and ventral fasciocutaneous penile skin flap) could be a great option in complex cases of penile urethral stricture.
